# Assessing communication skills during OSCE: need for integrated psychometric approaches

**DOI:** 10.1186/s12909-021-02552-8

**Published:** 2021-02-16

**Authors:** Giovanni Piumatti, Bernard Cerutti, Noëlle Junod Perron

**Affiliations:** 1 Division of Primary Care, Population Epidemiology Unit, Geneva University Hospitals, Geneva, Switzerland; 2 Institute of Public Health, Faculty of BioMedical Sciences, Università della Svizzera Italiana, Lugano, Switzerland; 3 Faculty of Medicine, Unit of Development and Research in Medical Education (UDREM), University of Geneva, Geneva, Switzerland; 4 Institute of Primary Care, Geneva University Hospitals, Geneva, Switzerland

**Keywords:** Communication assessment, OSCE, Item response theory, Measurement invariance

## Abstract

**Background:**

Physicians’ communication skills (CS) are known to significantly affect the quality of health care. Communication skills training programs are part of most undergraduate medical curricula and are usually assessed in Objective Structured Clinical Examinations (OSCE) throughout the curriculum. The adoption of reliable measurement instruments is thus essential to evaluate such skills.

**Methods:**

Using Exploratory Factor Analysis (EFA), Multi-Group Confirmatory Factor Analysis (MGCFA) and Item Response Theory analysis (IRT) the current retrospective study tested the factorial validity and reliability of a four-item global rating scale developed by Hodges and McIlroy to measure CS among 296 third- and fourth-year medical students at the Faculty of Medicine in Geneva, Switzerland, during OSCEs.

**Results:**

EFA results at each station showed good reliability scores. However, measurement invariance assessments through MGCFA across different stations (i.e., same students undergoing six or three stations) and across different groups of stations (i.e., different students undergoing groups of six or three stations) were not satisfactory, failing to meet the minimum requirements to establish measurement invariance and thus possibly affecting reliable comparisons between students’ communication scores across stations. IRT revealed that the four communication items provided overlapping information focusing especially on high levels of the communication spectrum.

**Conclusions:**

Using this four-item set in its current form it may be difficult to adequately differentiate between students who are poor in CS from those who perform better. Future directions in best-practices to assess CS among medical students in the context of OSCE may thus focus on (1) training examiners so to obtain scores that are more coherent across stations; and (2) evaluating items in terms of their ability to cover a wider spectrum of medical students’ CS. In this respect, IRT can prove to be very useful for the continuous evaluation of CS measurement instruments in performance-based assessments.

**Supplementary Information:**

The online version contains supplementary material available at 10.1186/s12909-021-02552-8.

## Background

Physicians’ communication skills (CS) are positively associated with a variety of health care outcomes, including patients’ physical and emotional health, pain control and better doctor-patient relationships [1–3]. CS are therefore recognized as crucial competencies for medical students and key components of medical practice [4–6]. Accordingly, nowadays most medical curricula include a longitudinal communication training track [7–9].

The Objective Structured Clinical Examination (OSCE) is a widely adopted tool by medical schools to assess CS among their students using interactions with standardized patients [10, 11]. It consists of different stations simulating real physician-patient encounters in clinical settings. CS can be assessed by OSCE stations specifically developed to test these skills as well as in an integrated way alongside other clinical skills [12]. The validity of students’ performances in CS strongly depends on the adopted measurement instrument. Two recent reviews evidenced a wide range of tools to measure medical students’ CS in OSCE [13, 14]. In addition to pointing out that such heterogeneity in measurement instruments prevents comparison across different settings, these reviews evidenced two main limitations of existing scales: the low inter-rater agreement and the lack of appropriate psychometric techniques in previous studies able to evaluate the overall validity and reliability of a scale. Since medical educators often need to evaluate students’ CS several times during the overall curriculum in order to monitor individual progresses and identify those in need of remediation [15], these same limitations become even more critical when comparing students’ longitudinal performances within the same institution.

As pointed out by Boon and Stewart [16], it is important that medical education educators and researchers focus on strengthening the evidence for the validity and reliability of available instruments in order to provide guidance for future assessments and to suggest how to improve existing scales. Although there is no unanimous consensus on this matter, previous researchers suggested that global rating scales for CS perform better than more complex behavioural checklists [13, 14, 16, 17]. However, when adopting global rating scales to measure CS we often make a priori assumptions about the factorial structure of these scales without actually testing them [13, 14]. In the context of OSCE, testing the validity of the factorial structure of a given global rating scale for CS means bringing evidence that this scale actually measures the same latent variable across different stations, namely CS. It also means ultimately assessing the invariance of this factorial structure across stations [18, 19]. More precisely, this implies that ideally when switching from a station to another the underlying model linking items to the latent variable is globally stable (*configural invariance*), the loadings (roughly speaking the contribution of every item to the CS) are stable (*metric invariance*), the intercepts are stable (i.e., there is no systematic response bias; *scalar invariance*), and the residual (i.e., the measure error) variability is also stable (*strict invariance*). Only by doing so we can test if we are reliably comparing scores between different stations on the basis of a derived global rating for CS. All these steps have important implications for the interpretation of CS’ scores within OSCE and for any conclusions about group- and station-related differences. In fact, unless we can conclude that the assumption of measurement invariance is met, we cannot legitimately compare scores across stations for example, as well as across contexts. For any global rating scale of CS thus, this technique can be used to assess whether a specific set of items is interpreted in a conceptually similar manner across stations dealing with different clinical scenarios.

Using multi-group factorial analysis techniques, the current study tested the measurement invariance of a widely adopted global rating scale proposed by Hodges and McIlroy [20–23] to assess medical students’ CS during OSCE. In addition, we further extended the psychometric assessment of this instrument into Item Response Theory analysis (IRT) [24]. The goal of IRT is first of all to assess the ‘ability’ of each item to tap into a specific portion of an underlying measure, in our case CS. Such items’ ability characteristic can be described as their ‘difficulty’, namely how hard it is for the examinee to score higher on a specific scale, given his or her overall level along the trait measured by that scale. Thus the IRT analysis allows to single out which items are able to differentiate between examinees with different levels of CS. More specifically, IRT can highlight if the items of this specific scale by Hodges and McIlroy provides overlapping information regarding medical students’ levels of CS. For example, it might happen that all items are good at differentiating between examinees with very high levels of CS and examinees with high levels, but none at differentiating between very high and low examinees for example. So, IRT would be able to point out directions for future improvement of the same instrument, suggesting the exclusion of items that do not add more information, or the inclusion of new items (or adaptation of the existing ones) to cover a wider spectrum of CS.

In sum, the dual approach of measurement invariance techniques and IRT, can bring evidence to help defining best-practice instruments, or suggesting modification of the existing ones, to measure CS during OSCE.

## Methods

### Sample and measures

This study was conducted at the Faculty of Medicine in Geneva, Switzerland, which offers a six-year curriculum to approximately 160 students per year. All the students take simultaneously a clinical skill exam of three OSCE stations of 18 min at the end of the third year, which focuses on history taking, physical exam, and a short explanation. Students also take an internal medicine and primary care medicine exam of six OSCE stations of 13 min, either at the mid or at the end of the fourth year, depending on the distribution of the clinical rotations. The clerkship in each discipline has a duration of two months. The stations (three in internal medicine and three in primary care) are integrative, and usually focus on history taking, physical exam, and case management. During the 2017/2018 academic year a global rating scale for CS [20] was added to the checklists of every station. Examiners were not formally trained to use this scale during OSCE at the Faculty of Medicine in Geneva, although 40% of them (*n* = 43) received formal training for its adoption during the Federal Licensing Examination in Switzerland. The scale has four dimensions measured on a 0 = *poor* to 4 = *excellent* Likert scale:

1. Respond to patient’s feelings and needs: Respond in a perspicacious and adequate way to solicitations and needs – verbal or non-verbal – of the patient.
2. Interview’s structure: Recognizable, coherent and flexible interaction plan during the entire consultation; the candidate conducts the interview in a coherent way.
3. Verbal expression: He/She communicates in an appropriate way that favours the comprehension and adapts the communication to the patient; the vocabulary is adapted to the socio-cultural level, with repetitions, summaries, articulations, tone, etc.
4. Non-verbal expression: He/She favours the relationship with the patient and his/her involvement through eye-contact, gestures, posture, interpersonal distance, pauses, etc.

In Switzerland, this scale is currently used in the context of the Federal Licensing Exam, where it showed good internal consistency among the four dimensions [25], as in other previous research within OSCE’s settings [20–23]. The selection of this instrument at the Swiss national level derives from few essential considerations: 1) the state-of-the-art of instructional methods and assessments for CS across Swiss medical schools; 2) the direct use of the instrument without the need for specific training; 3) the broad coverage of communication competencies being therefore applicable to different medical faculties independently by their curriculum; and 4) the ability to complete the assessment of CS in less than 2 min [25]. Nevertheless, to the best of the authors’ knowledge, no study has yet evaluated the invariance of the factorial structure of this scale across stations, or applied IRT to it. For the current study, we used CS evaluation scores for 147 fourth-year students across six stations and 149 third year students across three stations, for a total of 24 stations. As part of routine quality improvement projects no administrative permission was required to access the raw data used in the study. Specifically, this study was approved by the teaching committee and the anonymised data were provided by the administration.

### Data analysis

First, Exploratory Factor Analysis (EFA) with principal-component factor method was applied to the four-item set measuring CS in each single OSCE station. In this way we could explore the factorial structure of this instrument across different examiners’ scores. The following indexes were used to determine the best factorial solution: extraction of factors with eigenvalues > 1, total variance explained by the chosen factorial solution > 50%, factor loadings > 0.30, values from Kaiser-Meyer-Olkin test (KMO) regarding the suitability of the data for factor analyses > 0.7, significance of Bartlett’s test of sphericity to test the hypothesis of the multivariate normal distribution with zero covariance of the data, and Cronbach’s alphas reliability results > 0.70 [26–28].

Subsequently, we tested the assumption of measurement invariance for this four-item set across different OSCE stations (i.e., same students undergoing six or three stations) and across different groups of OSCE stations (i.e., different students undergoing groups of six or three stations) using Multi-Group Confirmatory Factor Analysis (MGCFA) with Maximum Likelihood (ML) estimation method. In order to test for measurement invariance across different groups of OSCE stations, we calculated students’ rounded mean scores for each item across stations so that each item had a single derived average score from different stations’ evaluations. Invariance testing followed a series of hierarchical models each adding an increasing number of constraints across groups [18]. First, it was tested whether the same parameters existed in the fourth-year students’ group undertaking six stations and in the third-year students’ groups undertaking three stations (*configural invariance*, that is the pattern of factor relationships are identical across groups). Then factor loadings were constrained to be equal across groups (*metric invariance*, that is the coefficient allowing to estimate the latent variable from the original score are identical), followed by item intercepts (*scalar invariance*, that is no systematic bias if one group is assessed rather than another), and residual variances (*strict invariance*, that is the error variability is the same whatever the group who is assessed). Model fit was evaluated using (1) *χ*^2^ goodness-of-fit, (2) Root Mean Square Error of Approximation (RMSEA; with values ≤ 0.08 being indicative of acceptable fit to the data), and (3) Comparative Fit Index (CFI; ≥ 0.9). Following recommendation from Chen [29], for testing configural invariance, a change of ≤ − 0.005 in CFI, supplemented by a change of ≥0.01 in RMSEA would indicate noninvariance; for testing metric or scalar invariance, a change of ≥ − 0.005 in CFI, supplemented by a change of ≥0.01 in RMSEA would indicate noninvariance.

Finally, IRT was used to examine single items’ properties and overall reliability of the scale on the whole sample. This technique allows to determine at which CS levels students are more likely to score at a given scale point. To do so, we used the items’ rounded mean scores for each student based on the scores from different stations that he or she had taken. Since all obtained rounded scores ranged between 2 and 4 with very small proportions of students who received a 2 on any given item (between 2 and 5%), we recoded every item into binary scores: 2 and 3 equal to 0 (i.e., failure) and 4 equal to 1 (i.e., success). This had implication for the type of IRT model to adopt, since with ordinal items we could have opted for a graded response model [30]. However, the very small percentage of individuals with averaged scores equal to 2 would have made inappropriate this approach for about 95% of our sample for which scores where either 3 or 4. Instead, working with binary items we tested two different IRT models: a 1-parameter model (1-PL), often called Rasch model, differentiating items based solely on their ‘difficulty’ (i.e., the parameter indicating at what level of the measured underlying trait a subject has to be to score higher on a specific item); and a 2-parameter model (2-PL) based on both item difficulty and item discrimination representing the extent to which an item discriminates between different trait levels (higher values indicating a stronger association with the measured construct) [31, 32]. We used a likelihood-ratio test to determine which one of these two models better explained our data. Items’ information functions were graphically plotted for interpretation along with the conditional standard errors and test information function for the four-item scale as a whole to evaluate the fit of the model to the data.

Analyses were conducted using Stata (version 15; StataCorp LP, College Station, TX, USA) and AMOS software (version 25.0; IBM Corp., Chicago, IL, USA).

## Results

Absolute values of skewness and kurtosis ranged respectively from 0 to 1.86, and from 1.09 to 9.72, suggesting that items’ scores were reasonably normally distributed (see Table A1 in the Appendix) [33]. The overall mean across all stations and students were respectively 3.07 (81.3% of scoring 3 or 4) for Item 1, 3.17 (83.7% of 3 or 4) for Item 2, 3.24 (87.4% of 3 or 4) for Item 3, and 3.16 (83.7% of 3 or 4) for Item 4. Correlations between the four items ranged between 0.20 and 0.84 across all stations.

Results from EFA suggested that across all stations the one-factor solution appeared to be the most appropriate to synthesize the four global rating items, with factor loadings ranging from 0.56 to 0.94 and Cronbach’s alphas ranging from 0.69 to 0.92 (Table 1). Results of the MGCFA are summarised in Table 2. Only the observations from the first group of students can be considered reasonably invariant across stations, although the one-factor solution for CS in this group only reached the threshold for *partial* invariance as described by Byrne et al. [34]. Reading from the results reported in Table A2, also in the case of measurement invariance testing across different groups of OSCE stations (i.e., different students undergoing groups of six or three stations) the minimum requirement for measurement invariance was not reached. More specifically, we cannot safely assume that the calibration of these items is similar across stations. This implies that the meaning of the obtained score from this scale is not the same across stations [18].

**Table 1:** Results of exploratory factor analyses applied to the four-item communication scale by OSCE stations: Standardized results from one-factor solutions are shown

Station	One-factor				Variance explained ^a^	KMO	Bartlett’s *p*	*α*
	Item 1	Item 2	Item 3	Item 4				
	*β*	*β*	*β*	*β*				
Group 1 (*n* = 35)								
Station 1	0.841	0.846	0.904	0.864	75%	0.826	< 0.001	0.880
Station 2	0.879	0.680	0.747	0.935	67%	0.716	< 0.001	0.827
Station 3	0.787	0.741	0.789	0.596	54%	0.719	0.001	0.688
Station 4	0.922	0.853	0.866	0.848	76%	0.747	< 0.001	0.894
Station 5	0.844	0.749	0.826	0.816	66%	0.667	< 0.001	0.824
Station 6	0.844	0.903	0.684	0.906	70%	0.797	< 0.001	0.854
Group 2 (*n* = 51)								
Station 1	0.857	0.562	0.914	0.914	68%	0.768	< 0.001	0.828
Station 2	0.828	0.831	0.879	0.868	73%	0.800	< 0.001	0.873
Station 3	0.874	0.854	0.929	0.933	81%	0.845	< 0.001	0.916
Station 4	0.860	0.661	0.829	0.872	66%	0.786	< 0.001	0.818
Station 5	0.760	0.761	0.912	0.829	67%	0.681	< 0.001	0.820
Station 6	0.711	0.682	0.808	0.737	54%	0.608	< 0.001	0.711
Group 3 (*n* = 61)								
Station 1	0.819	0.708	0.903	0.861	68%	0.778	< 0.001	0.841
Station 2	0.857	0.777	0.829	0.786	66%	0.771	< 0.001	0.825
Station 3	0.723	0.695	0.819	0.852	60%	0.763	< 0.001	0.775
Station 4	0.770	0.625	0.789	0.797	56%	0.662	< 0.001	0.730
Station 5	0.706	0.800	0.858	0.823	64%	0.704	< 0.001	0.808
Station 6	0.804	0.782	0.836	0.820	66%	0.808	< 0.001	0.821
Group 4 (*n* = 89)								
Station 1	0.866	0.809	0.859	0.846	71%	0.823	< 0.001	0.865
Station 2	0.848	0.826	0.874	0.910	75%	0.796	< 0.001	0.885
Station 3	0.900	0.697	0.836	0.846	68%	0.767	< 0.001	0.838
Group 5 (*n* = 60)								
Station 1	0.813	0.709	0.761	0.874	63%	0.724	< 0.001	0.798
Station 2	0.758	0.846	0.825	0.857	68%	0.774	< 0.001	0.838
Station 3	0.777	0.841	0.874	0.801	68%	0.803	< 0.001	0.842

Notes. ^a^ Variance explained by one single factor with eigenvalue > 1; *β*: Standardized factor loadings; KMO: Kaiser-Meyer-Olkin; *α*: Cronbach’s alpha; Item 1: Respond to patient’s feelings and needs: respond in a perspicacious and adequate way to solicitations and needs – verbal or non-verbal – of the patient; Item 2: Interview’s structure: recognizable, coherent and flexible interaction plan during the entire consultation; the candidate conducts the interview in a coherent way; Item 3: Verbal expression: he/she communicates in an appropriate way that favours the comprehension and adapts the communication to the patient; the vocabulary is adapted to the socio-cultural level, with repetitions, summaries, articulations, tone, etc.; Item 4: Non-verbal expression: he/she favours the relationship with the patient and his/her involvement trough eye-contact, gestures, posture, interpersonal distance, pauses, etc.

**Table 2:** Results of the four-item communication scale measurement invariance testing across OSCE stations

Model	*χ*^2^	*df*	RMSEA (90% CIs)	CFI	Δ*χ*^2^	Δ*df*	ΔRMSEA	ΔCFI	Comparison
Group 1 (*n* = 35)									
Model 1. Configural invariance	23.66^*^	12	0.069 (0.025, 0.110)	0.967					
Model 2. Metric invariance	41.72^*^	27	0.052 (0.025, 0.110)	0.959	18.06	15	−0.017	−0.008	Model 2 vs. Model 1
**Model 3. Scalar invariance**	73.27^**^	**47**	**0.052 (0.027, 0.075)**	**0.926**	31.55^*^	**20**	**0.000**	**−0.033**	**Model 3 vs. Model 2**
Model 4. Strict invariance	159.89^***^	67	0.082 (0.066, 0.099)	0.739	86.62^***^	20	0.030	−0.187	Model 4 vs. Model 3
Group 2 (*n* = 51)									
Model 1. Configural invariance	24.72^*^	12	0.059 (0.025, 0.093)	0.977					
**Model 2. Metric invariance**	54.18^**^	**27**	**0.058 (0.035, 0.080)**	**0.950**	29.46^*^	**15**	**−0.001**	**−0.027**	**Model 2 vs. Model 1**
Model 3. Scalar invariance	124.41^***^	47	0.074 (0.058, 0.090)	0.859	70.23^***^	20	0.016	−0.091	Model 3 vs. Model 2
Model 4. Strict invariance	150.36^***^	67	0.064 (0.051, 0.078)	0.848	25.95	20	−0.010	−0.011	Model 4 vs. Model 3
Group 3 (*n* = 61)									
Model 1. Configural invariance	22.48^*^	12	0.049 (0.014, 0.080)	0.978					
**Model 2. Metric invariance**	41.61^*^	**27**	**0.039 (0.010, 0.061)**	**0.969**	**19.13**	**15**	**−0.010**	**−0.009**	**Model 2 vs. Model 1**
Model 3. Scalar invariance	105.10^***^	47	0.059 (0.044, 0.074)	0.875	63.49^***^	20	0.020	−0.094	Model 3 vs. Model 2
Model 4. Strict invariance	171.10^***^	67	0.066 (0.054, 0.078)	0.777	66.00^***^	20	0.007	−0.098	Model 4 vs. Model 3
Group 4 (*n* = 89)									
**Model 1. Configural invariance**	**9.57**	**6**	**0.047 (0.000, 0.101)**	**0.993**					
Model 2. Metric invariance	25.69^*^	12	0.066 (0.030, 0.101)	0.973	16.12^*^	6	0.019	−0.020	Model 2 vs. Model 1
Model 3. Scalar invariance	45.83^***^	20	0.070 (0.043, 0.097)	0.950	20.14^**^	8	0.004	−0.023	Model 3 vs. Model 2
Model 4. Strict invariance	74.09^***^	28	0.079 (0.057, 0.101)	0.910	28.26^***^	8	0.009	−0.040	Model 4 vs. Model 3
Group 5 (*n* = 60)									
Model 1. Configural invariance	8.38	6	0.047 (0.000, 0.116)	0.991					
**Model 2. Metric invariance**	22.27^*^	**12**	**0.070 (0.018, 0.114)**	**0.960**	13.89^*^	**6**	**0.023**	**0.031**	**Model 2 vs. Model 1**
Model 3. Scalar invariance	46.17^***^	20	0.086 (0.053, 0.119)	0.898	23.90^**^	8	0.016	−0.062	Model 3 vs. Model 2
Model 4. Strict invariance	53.33^**^	28	0.071 (0.041, 0.100)	0.902	7.16	8	0.005	0.004	Model 4 vs. Model 3

Notes. *χ*^2^ Chi-square goodness of fit, *df* degrees of freedom, *RMSEA* Root Mean Square Error of Approximation, *90% CIs* 90% Confidence Intervals for RMSEA, *CFI* Comparative Fit Index, *Δχ* Chi-square goodness of fit difference, Δ*df* degrees of freedom difference, *ΔCFI* CFI difference, *ΔRMSEA* RMSEA difference. ^*^p < .05, ^**^p < .01, ^***^p < .001. The best fitting solutions for each group are marked in bold

**Fig. 1:**
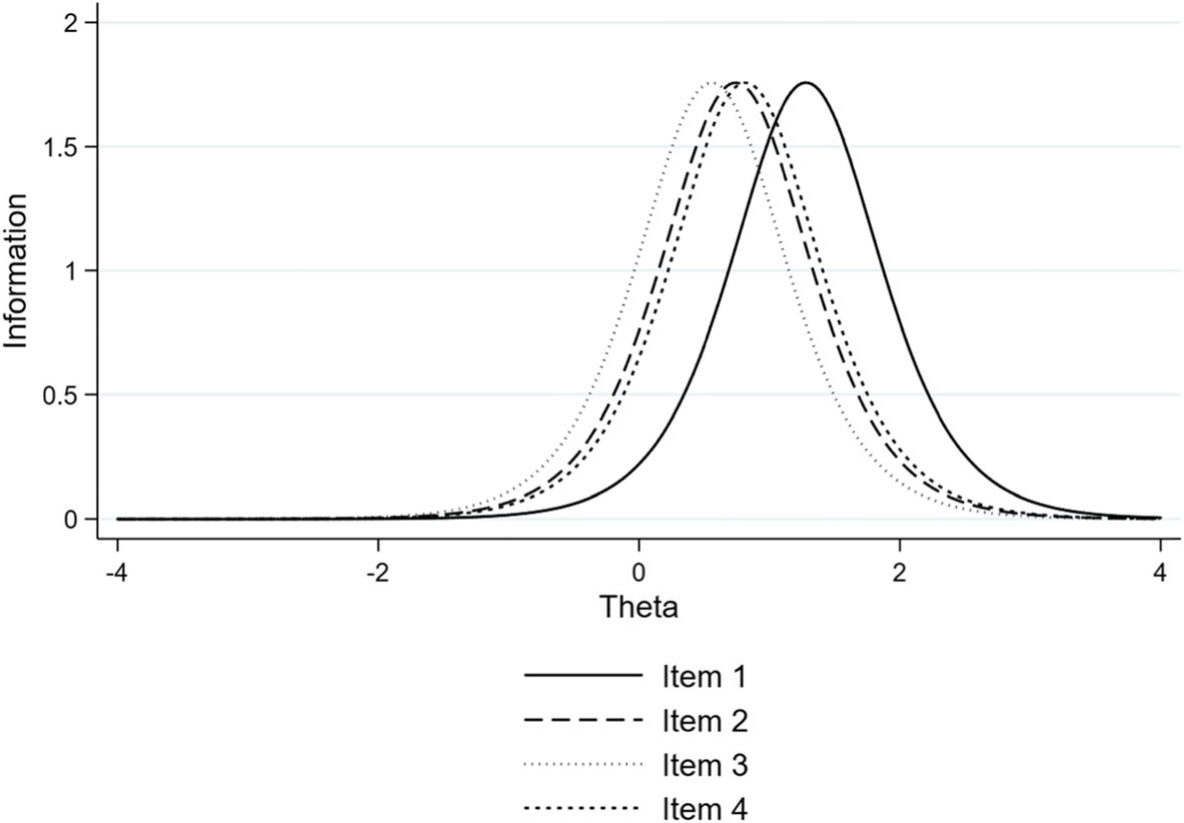
Item information graph for Rasch (1-PL) analysis of four-item communication scale (*N* = 296). Notes. Item 1: Respond to patient’s feelings and needs: respond in a perspicacious and adequate way to solicitations and needs – verbal or non-verbal – of the patient; Item 2: Interview’s structure: recognizable, coherent and flexible interaction plan during the entire consultation; the candidate conducts the interview in a coherent way; Item 3: Verbal expression: he/she communicates in an appropriate way that favours the comprehension and adapts the communication to the patient; the vocabulary is adapted to the socio-cultural level, with repetitions, summaries, articulations, tone, etc.; Item 4: Non-verbal expression: he/she favours the relationship with the patient and his/her involvement trough eye-contact, gestures, posture, interpersonal distance, pauses, etc.

**Fig. 2:**
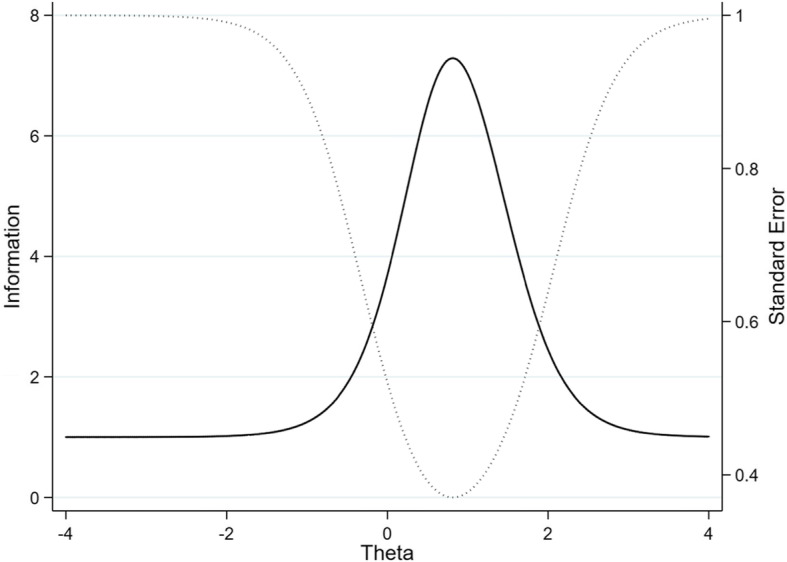
Information graph showing the four-item communication scale: Test information function (solid line) and conditional standard error curve (dotted line) (*N* = 296)

There was no evidence of a difference between the two-parameter IRT model and the one-parameter model (*χ*^2^ = 3.30, *df* = 3, likelihood ratio test *p* = 0.348). This suggested that there is no difference in how discriminating the four items are, but the items can be differentiated based solely on their difficulty. Item 1 (Response to the patient’s feeling and need) appeared to be the most difficult (see Table 3 and Fig. 1). An examinee must have a latent score (i.e., Theta) equal to 1.28 to get a 50% chance to obtain the highest mark for this item. Theta represents the true latent score in CS that has been standardized on a scale from − 4 to 4, although it is unlikely to find somebody scoring at those extremes. Accordingly, a student who has a very low ability in CS, say Theta = − 2, would have a very small probability of getting a high score on this item. Conversely, a student with high ability in CS, say Theta = 2, would most certainly get a high score on this item. Figure 1 shows the amount of information brought by each item. Items 2, 3 and 4 appear to provide overlapping information and are thus redundant (items 2 and 4 in particular). Figure 2 depicts the conditional standard errors and test information function for the four-item communication scale. This battery of items seems to have low levels of standard error and concurrently a high level of provided information approximately only for latent scores ranging from 0 to 2.

**Table 3:** Results of a 1-parameter (i.e., difficulty) Rasch model applied to the four-item communication scale (*N* = 296)

Item	Coefficient	Standard error	(90% CIs)
1. Respond to patient’s feelings and needs: respond in a perspicacious and adequate way to solicitations and needs – verbal or non-verbal – of the patient.	1.28	0.12	(1.08, 1.48)
2. Interview’s structure: recognizable, coherent and flexible interaction plan during the entire consultation; the candidate conducts the interview in a coherent way.	0.74	0.09	(0.59, 0.90)
3. Verbal expression: he/she communicates in an appropriate way that favours the comprehension and adapts the communication to the patient; the vocabulary is adapted to the socio-cultural level, with repetitions, summaries, articulations, tone, etc.	0.56	0.09	(0.41, 0.70)
4. Non-verbal expression: he/she favours the relationship with the patient and his/her involvement trough eye-contact, gestures, posture, interpersonal distance, pauses, etc.	0.82	0.10	(0.66, 0.98)

## Discussion

This study tested the factorial validity and reliability of a four-item global scale to assess medical students’ CS in OSCE settings. When considering each specific OSCE station separately, it appears that this scale provides reliable information about students’ CS. However, the assumption of measurement invariance across stations was clearly not met, suggesting that there is likely to be both little agreement between different examiners and a significant interaction between the examinee and the examination settings. Thus the comparisons of students’ CS across different OSCE stations is difficult. For example, it may be problematic even interpreting mean differences in CS between different stations, as well as reliably comparing scores of the same students across time. Indeed, configural and metric invariances for the whole factor structure and for each factor loading are crucial for the interpretation of the constructs and are requisites for all other tests [18]. Based on these findings, we cannot ensure that this four-item communication scale is invariant across stations, not even when students are evaluated along three instead of six OSCE. This imposes serious challenges if we want to compare in a meaningful way CS between different simulated clinical scenarios.

Previous studies have noticed that the main sources of variance in CS scores during OSCE are related to candidates’ individual characteristics such as level of training that become evident across different stations, but also to examiners’ propensity to pass or fail candidates [20, 35, 36]. More actions would be therefore needed to ‘normalise’ assessments across examiners and stations. For instance, it is advisable to revise with the examiners how to adequately use the entire 5-level Likert scale for each item. In this regard, given the fact that the lowest levels of the rating scale were extremely unlikely to be used, revisions of the instrument may raise the question of reducing the scoring range to binary options (e.g., pass/fail). In addition, as an alternative to a global score in CS, future adoptions of this instrument may use the scores from single items to compare students’ performances between stations rather than computing a summated outcome for CS based on this four-item set.

The IRT analysis brought important evidence about how this set of items may be improved in the future, for example by elaborating items that would better discriminate between very poor and lower-average communication skilled medical students. Thus, adding new items tapping into the uncovered portion of the CS spectrum, or modifying existing ones accordingly, may help to better discriminate between students and avoid ceiling effects in the scoring system, with every student scoring high on communication. On a related note, shorter versions of this current scale should also be tested, since the two items measuring interview’s structure and non-verbal expression appear to measure the same portion of the CS spectrum and are thus redundant. These results indicate that to improve the discriminative power of this scale we may want to add more items or modify existing ones so to tap into low levels of CS as in this current form we are not able to adequately differentiates between students who have low levels of CS from those who perform better.

It is also important to remind that if we interpret the poor measurement invariance performance of this global rating scale in relation to a scarce interrater reliability, this may be related to several different factors not assessed in the current study. For example, station duration has been reported to affect performance assessment reliability [13, 37]. Moreover, the same interpretation of the terminology used by a given instrument can play a role when adequate agreement of the empirical indicators in relation with the underlying concepts is not met between reviewers [38, 39]. Finally, we pointed out that no unanimous consensus exists when it comes to preferences for global rating scales for CS vs. more complex behavioural checklists [13, 14, 16, 17]. Although brief instruments may less likely fail to meet psychometric standards for comparability due to the shorter number of parameters to estimate, it remains an open question whether these competencies can be assessed by a unique scale across different specialties and practice conditions [40]. As suggested by Setyonugroho et al. [13] and Cömert et al. [14], in addition to investing on the standardisation and evaluation of CS’ assessment tools, medical schools should match any measure of CS with how this concept is taught along their undergraduate and postgraduate training programs.

In sum, while our results evidence the limitations of this four-item global scale for CS in terms of comparability across stations and discriminant ability between students, in accordance with previous research [41], it showed good reliability results within OSCE stations. Accordingly, the joint use of measurement invariance techniques and IRT provide knowledge to improve its adoption in future assessments.

### Limitations and future research

The major limitations of the current study are related to the items’ scores averaging across stations posing a threat to the IRT’s assumptions of unidimensionality and local independence, and the relatively small sample size. In fact, although the analyses were run on the original items’ scores for testing the measurement invariance across OSCE stations, an approximation had to be done for the use of the IRT in order to maximise the available data and obtain a sample size closer to what is considered acceptable for Rasch modeling [24]. On a related note, De Champlain [42] pointed out that IRT might not be particularly well suited to OSCEs since students’ performances on the same skills may vary across different stations’ domains covering specific clinical skills. Future studies may thus adopt this type of analytical approach to estimate items’ performances in single OSCE stations or clinical scenarios, provided that they have adequate sample sizes. For what concerns sample size, this was linked to the retrospective nature of the study can certainly been overcome in future research. Although recommendations about adequate sample size in the context of factor analysis vary from as low as 5 cases per estimated parameter [43], to 10 [44] or 20 [33], it has also been shown how sample size does not significantly affect hypotheses testing of measurement invariance [45]. Finally, future studies can be designed for examining how the set of items analysed here may vary in their measurement invariance properties across examiners grouped by specific characteristics (e.g., gender, specialty, OSCE evaluating experience).

## Conclusions

The current study showed the advantages of adopting statistical approaches such as multi-group factorial analysis and IRT to evaluate a global rating scale for assessing CS among undergraduate medical students during OSCE. Our results and approach may help medical educators to normalize efforts across settings and institutions and create guidelines for the evaluation and adoption of measurement instruments for CS such as the one tested here. As pointed out by previous research [35, 36, 42, 46–49], analytical approaches such as IRT modeling can prove to be very useful in medical education especially for what concerning performance-based assessments. On the basis of this type of analysis, medical schools can support continuous evaluations of their assessment tools so to evidence where to improve them and propose new best-practices from evidence-based research.

## Additional file

**Additional file 1: Table A1**. Descriptive statistics at the item-level for the communication scale by OSCE stations. **Table A2**. Results of the four-item communication scale measurement invariance testing across examination groups

## Data Availability

The datasets generated and/or analysed during the current study are not publicly available due to the privacy of the students but are available from the corresponding author on reasonable request.
