# Supplementary material for: Assessing communication skills during OSCE: need for integrated psychometric approaches
Source: BMC Med Educ. 2021 Feb 16;21:106. doi: 10.1186/s12909-021-02552-8 (PMC7887794; doi:10.1186/s12909-021-02552-8)
Supplement: Supplementary file 1 — Additional file 1: Table A1. Descriptive statistics at the item-level for the communication scale by OSCE stations. Table A2. Results of the four-item communication scale measurement invariance testing across examination groups [file 12909_2021_2552_MOESM1_ESM.docx]

**Appendix**

**Assessing communication skills during OSCE: Need for integrated psychometric approaches**

Table A1. Descriptive statistics at the item-level for the communication scale by OSCE stations

Table A2. Results of the four-item communication scale measurement invariance testing across examination groups

Table A1. Descriptive statistics at the item-level for the communication scale by OSCE stations

| Station | Item 1 | | | | Item 2 | | | | Item 3 | | | | Item 4 | | | |
| --- | --- | --- | --- | --- | --- | --- | --- | --- | --- | --- | --- | --- | --- | --- | --- | --- |
|  | M | SD | Range | %“4” | M | SD | Range | %“4” | M | SD | Range | %“4” | M | SD | Range | %“4” |
| Group 1 (n = 35) |  |  |  |  |  |  |  |  |  |  |  |  |  |  |  |  |
| Station 1 | 3.06 | 0.59 | 1 – 4 | 17 | 2.91 | 0.70 | 2 – 4 | 20 | 3.06 | 0.68 | 2 – 4 | 26 | 2.91 | 0.74 | 1 – 4 | 20 |
| Station 2 | 2.80 | 0.58 | 2 – 4 | 9 | 2.91 | 0.61 | 2 – 4 | 11 | 3.00 | 0.54 | 2 – 4 | 14 | 2.83 | 0.62 | 2 – 4 | 11 |
| Station 3 | 3.09 | 0.56 | 2 – 4 | 20 | 3.00 | 0.59 | 2 – 4 | 17 | 3.17 | 0.51 | 2 – 4 | 23 | 3.00 | 0.73 | 0 – 4 | 17 |
| Station 4 | 2.91 | 0.56 | 2 – 4 | 11 | 2.86 | 0.55 | 2 – 4 | 9 | 3.03 | 0.62 | 2 – 4 | 20 | 3.00 | 0.59 | 2 – 4 | 17 |
| Station 5 | 3.06 | 0.59 | 2 – 4 | 20 | 3.17 | 0.51 | 2 – 4 | 23 | 3.31 | 0.53 | 2 – 4 | 34 | 3.26 | 0.56 | 2 – 4 | 31 |
| Station 6 | 3.23 | 0.97 | 0 – 4 | 49 | 3.26 | 0.92 | 0 – 4 | 49 | 2.94 | 0.97 | 0 – 4 | 31 | 3.23 | 0.94 | 0 – 4 | 46 |
| Group 2 (n = 51) |  |  |  |  |  |  |  |  |  |  |  |  |  |  |  |  |
| Station 1 | 3.20 | 0.87 | 1 – 4 | 45 | 3.41 | 0.70 | 2 – 4 | 53 | 3.33 | 0.95 | 1 – 4 | 57 | 3.55 | 0.64 | 2 – 4 | 63 |
| Station 2 | 2.90 | 0.76 | 1 – 4 | 22 | 2.80 | 0.83 | 1 – 4 | 20 | 3.10 | 0.78 | 1 – 4 | 33 | 3.02 | 0.76 | 1 – 4 | 27 |
| Station 3 | 2.92 | 1.11 | 0 – 4 | 41 | 3.20 | 0.98 | 0 – 4 | 49 | 3.25 | 0.93 | 1 – 4 | 51 | 3.18 | 0.93 | 1 – 4 | 47 |
| Station 4 | 3.04 | 0.69 | 1 – 4 | 24 | 2.98 | 0.71 | 2 – 4 | 24 | 3.10 | 0.64 | 2 – 4 | 25 | 3.10 | 0.64 | 2 – 4 | 25 |
| Station 5 | 3.47 | 0.61 | 2 – 4 | 53 | 3.51 | 0.76 | 1 – 4 | 65 | 3.61 | 0.57 | 2 – 4 | 65 | 3.63 | 0.56 | 2 – 4 | 67 |
| Station 6 | 2.80 | 0.66 | 2 – 4 | 14 | 2.98 | 0.68 | 1 – 4 | 20 | 3.16 | 0.61 | 2 – 4 | 27 | 3.04 | 0.56 | 2 – 4 | 18 |
| Group 3 (n = 61) |  |  |  |  |  |  |  |  |  |  |  |  |  |  |  |  |
| Station 1 | 2.61 | 1.04 | 0 – 4 | 20 | 2.95 | 0.78 | 1 – 4 | 26 | 2.95 | 0.90 | 0 – 4 | 26 | 2.85 | 0.89 | 1 – 4 | 23 |
| Station 2 | 2.98 | 0.59 | 1 – 4 | 15 | 3.10 | 0.70 | 1 – 4 | 26 | 2.97 | 0.68 | 1 – 4 | 18 | 2.90 | 0.62 | 1 – 4 | 11 |
| Station 3 | 2.97 | 0.77 | 1 – 4 | 25 | 3.28 | 0.73 | 1 – 4 | 41 | 3.26 | 0.70 | 2 – 4 | 41 | 3.16 | 0.90 | 0 – 4 | 41 |
| Station 4 | 3.30 | 0.56 | 2 – 4 | 34 | 3.41 | 0.69 | 2 – 4 | 52 | 3.39 | 0.69 | 2 – 4 | 51 | 3.33 | 0.70 | 2 – 4 | 28 |
| Station 5 | 3.03 | 0.63 | 1 – 4 | 20 | 2.92 | 0.71 | 1 – 4 | 18 | 3.11 | 0.64 | 1 – 4 | 25 | 3.08 | 0.71 | 1 – 4 | 26 |
| Station 6 | 3.34 | 0.66 | 2 – 4 | 44 | 3.43 | 0.50 | 3 – 4 | 43 | 3.44 | 0.53 | 2 – 4 | 46 | 3.36 | 0.63 | 2 – 4 | 44 |
| Group 4 (n = 89) |  |  |  |  |  |  |  |  |  |  |  |  |  |  |  |  |
| Station 1 | 3.18 | 0.78 | 2 – 4 | 40 | 3.36 | 0.66 | 2 – 4 | 46 | 3.38 | 0.65 | 2 – 4 | 47 | 3.39 | 0.70 | 1 – 4 | 51 |
| Station 2 | 3.25 | 0.63 | 2 – 4 | 35 | 3.26 | 0.75 | 1 – 4 | 43 | 3.39 | 0.67 | 2 – 4 | 49 | 3.21 | 0.67 | 2 – 4 | 35 |
| Station 3 | 3.15 | 0.79 | 1 – 4 | 36 | 3.18 | 0.75 | 1 – 4 | 37 | 3.25 | 0.70 | 2 – 4 | 39 | 3.00 | 0.84 | 1 – 4 | 31 |
| Group 5 (n = 60) |  |  |  |  |  |  |  |  |  |  |  |  |  |  |  |  |
| Station 1 | 3.13 | 0.65 | 1 – 4 | 27 | 3.03 | 0.69 | 1 – 4 | 22 | 3.25 | 0.60 | 1 – 4 | 32 | 3.10 | 0.73 | 1 – 4 | 28 |
| Station 2 | 2.83 | 0.69 | 1 – 4 | 13 | 3.27 | 0.82 | 1 – 4 | 45 | 3.33 | 0.66 | 2 – 4 | 43 | 3.13 | 0.72 | 2 – 4 | 33 |
| Station 3 | 3.23 | 0.62 | 2 – 4 | 33 | 3.40 | 0.69 | 2 – 4 | 52 | 3.38 | 0.67 | 2 – 4 | 48 | 3.25 | 0.68 | 2 – 4 | 38 |

Notes. Item 1: Respond to patient’s feelings and needs: respond in a perspicacious and adequate way to solicitations and needs – verbal or non-verbal – of the patient; Item 2: Interview’s structure: recognizable, coherent and flexible interaction plan during the entire consultation; the candidate conducts the interview: in a coherent way; Item 3: Verbal expression: he/she communicates in an appropriate way that favours the comprehension and adapts the communication to the patient; the vocabulary is adapted to the socio-cultural level, with repetitions, summaries, articulations, tone, etc.; Item 4: Non-verbal expression: he/she favours the relationship with the patient and his/her involvement trough eye-contact, gestures, posture, interpersonal distance, pauses, etc.;

%“4”: percentages of scores equal to “4”

Table A2. Results of the four-item communication scale measurement invariance testing across examination groups (i.e., different students undergoing groups of six or three stations)

| Model | *χ*^2^ | *df* | RMSEA (90% CIs) | CFI | Δ*χ*^2^ | Δ*df* | ΔRMSEA | ΔCFI | Comparison |
| --- | --- | --- | --- | --- | --- | --- | --- | --- | --- |
| **Model 1. Configural invariance** | **14.34** | **10** | **0.039 (0.000, 0.080)** | **0.993** |  |  |  |  |  |
| Model 2. Metric invariance | 48.65^***^ | 20 | 0.070 (0.045, 0.095) | 0.955 | 34.31^***^ | 10 | 0.031 | -0.038 | Model 2 vs. Model 1 |
| Model 3. Scalar invariance | 81.06^***^ | 32 | 0.073 (0.053, 0.092) | 0.924 | 32.41^**^ | 12 | 0.003 | -0.031 | Model 3 vs. Model 2 |
| Model 4. Strict invariance | 118.48^***^ | 44 | 0.076 (0.060, 0.093) | 0.884 | 37.42^***^ | 12 | 0.003 | -0.040 | Model 4 vs. Model 3 |

Notes. *χ*^2^: Chi-square goodness of fit; *df*: degrees of freedom; RMSEA: Root Mean Square Error of Approximation; 90% CIs: 90% Confidence Intervals for RMSEA; CFI: Comparative Fit Index; Δ*χ*^2^: Chi-square goodness of fit difference; Δ*df*: degrees of freedom difference; ΔCFI: CFI difference; ΔRMSEA: RMSEA difference. ^*^*p <* .05, ^**^*p <* .01, ^***^*p <* .001. The best fitting solution is marked in bold
